# Impact of lifestyle and psychological resilience on survival among the oldest-old in China: a cohort study

**DOI:** 10.3389/fpubh.2023.1329885

**Published:** 2023-12-19

**Authors:** Jianping Cai, Yumeng Gao, Tingfa Hu, Lv Zhou, Hongye Jiang

**Affiliations:** Department of Medical Insurance, Jinshan Hospital of Fudan University, Shanghai, China

**Keywords:** lifestyle, psychological resilience, survival, mediation, moderation, oldest-old

## Abstract

**Introduction:**

Healthy lifestyles and psychological resilience are important factors influencing the life expectancy of the oldest-old (≥80 years). Stratified by urban and rural groups, this study used a 10-year cohort to examine the mechanism of lifestyle and psychological resilience on the survival of the oldest-old in China.

**Methods:**

This study used the China Longitudinal Healthy Longevity Survey datasets spanning from 2008 to 2018, and 9,250 eligible participants were included. The primary outcome variable was all-cause mortality, and independent variables included healthy lifestyle index and psychological resilience. Six covariates were included in the survival analysis and moderation-mediation model, such as gender and annual household income.

**Results:**

This study found that the oldest-old with five healthy lifestyles had the longest survival time, averaging 59.40 months for urban individuals and 50.08 months for rural individuals. As the lifestyle index increased, the survival rate significantly increased. The Cox regression showed that for the urban oldest-old, the lifestyle index served as a protective factor for survival outcomes. However, this effect lost statistical significance among rural oldest-old individuals. For urban oldest-old individuals, psychological resilience significantly mediated and moderated the effect of the lifestyle index on survival status, but the moderating effect was not statistically significant for the rural ones.

**Discussion:**

Overall, healthy lifestyles and psychological resilience can be effective in enhancing the survival of the oldest-old, and there are differences between urban and rural population, so different interventions should be adopted for urban and rural areas to achieve longer life in China.

## 1 Introduction

According to the ‘Statistical Bulletin on the Development of China’s Health in 2021,’ the life expectancy of Chinese residents increased from 77.93 years in 2020 to 78.2 years in 2021. This indicates a growing population of the oldest-old, referring to people aged 80 years and over. By 2035, the life expectancy in China is expected to reach 81.3 years, with female life expectancy likely to exceed 90 years in developed provinces and cities (1). To support the health and well-being of the oldest-old, China will face various challenges, including caregiving, healthcare, and social support (2, 3). Therefore, exploring ways to enhance their health-related behaviors and psychology in late life and ensure a longer life for the oldest-old is a matter of significant concern.

Lifestyle behaviors are an important factor affecting the quality of life, individual overall health, and participation in social activities, especially for the oldest-old. Life satisfaction is an important index to comprehensively evaluate people’s living conditions, and a national survey in China found that lifestyle was positively related to the life satisfaction of older people (4). Additionally, embracing an appropriate lifestyle is crucial for overall well-being, with factors like diet, exercise, and other habits playing pivotal roles in maintaining good health. Previous studies showed that improvements in nutrition were beneficial for older adults to prevent, modulate, or ameliorate many age-related diseases and conditions (5). Previous studies also suggested that regular physical exercise in the oldest-old contributed to the increase or maintenance of muscle function, which enables them to engage in more social activities, thereby enhancing their overall health conditions (6). There is a consensus that smoking and alcohol consumption are common risk factors for physical health across various populations. Besides, previous studies also indicated that smoking and alcohol consumption were associated with the cognitive function, anxiety, and depression of the oldest-old (7, 8). However, there is currently a lack of longitudinal studies to validate the relationship between lifestyle and psychological resilience in the oldest-old, which is crucial for their survival outcomes.

Psychological resilience refers to an individual’s ability to maintain a positive attitude and adapt to stresses, challenges, and adversities (9). Good psychological resilience could help maintain the health of the oldest-old, and enable them to strengthen their motivation to cope with various problems and challenges. A nationwide longitudinal study showed that higher levels of psychological resilience were significantly associated with reduced risk of becoming activities of daily living disabled in the oldest-old (10). Additionally, previous studies also showed that psychological resilience played crucial roles in addressing problems related to basic self-care, self-health management, and seeking social support among the oldest-old (11–13). However, there is a lack of research on the relationship between psychological resilience and survival status in the oldest-old.

Both healthy lifestyle choices and positive psychological resilience could be critical determinants of survival outcomes among the oldest-old. Previous studies have shown that maintaining healthy lifestyle practices such as physical activity, tea consumption, and abstaining from smoking and alcohol consumption contributed to improving the survival status of the oldest-old (14, 15). Besides, studies have suggested that the oldest-old, as a vulnerable group, often faced various problems of mental health, including deficits in psychological resilience (16, 17). Psychological resilience may facilitate the motivation of the oldest-old to cope with health-related risk factors and is essential for maintaining their survival status. Therefore, we propose the following research hypotheses:

*H1:* Healthy lifestyle is directly associated with the survival of the oldest-old.

*H2:* Psychological resilience is directly associated with the survival of the oldest-old.

Lifestyle should be recognized as a common factor influencing both the physical and psychological health of the oldest-old. Previous study has revealed that unhealthy habits such as smoking and excessive alcohol consumption were risk factors for various chronic diseases, including cardiovascular and cerebrovascular diseases (18). Furthermore, previous studies have also shown that lifestyles could significantly influence psychological resilience in different groups of people. Healthy lifestyles have been proven to be associated with mental health among cancer survivors, and combinations of higher healthy lifestyles and better mental health were associated with their decreased mortality (19). Besides, a previous study showed that healthy lifestyles were beneficial to improve successful cognitive aging among middle-aged and older community residents (20). However, there is currently a lack of empirical research examining the relationship between lifestyle factors, psychological resilience, and survival status among the oldest-old. Adopting a healthy lifestyle can help foster positive cognitive beliefs, enhance psychological resilience among the oldest-old, and improve their life expectancy. Hence, we proposed the following hypothesis:

*H3:* Psychological resilience mediates the relationship between lifestyle and survival of the oldest-old.

The oldest-old often faced with health problems such as chronic illness and declining physical function. Psychological resilience can help them in maintaining a positive outlook on life, and it will help them implement an active and optimistic lifestyle to deal with a variety of health challenges (10), which may ultimately help extend their chances of survival. Additionally, a previous study indicated that healthy and various eating habits were often closely associated with good psychological resilience in older adults (11), so healthy eating plus psychological resilience could contribute to the longevity to a greater extent. Good psychological resilience is an important basis for all behaviors of the older adult/adults, which can provide intrinsic motivation for their behaviors and strengthen the effect of healthy behaviors. (11, 21). Ultimately, this is conducive to improving the overall survival of the oldest-old. Therefore, we proposed the hypothesis:

*H4:* Psychological resilience moderates the relationship between lifestyle and survival of the oldest-old.

**Figure 1:**
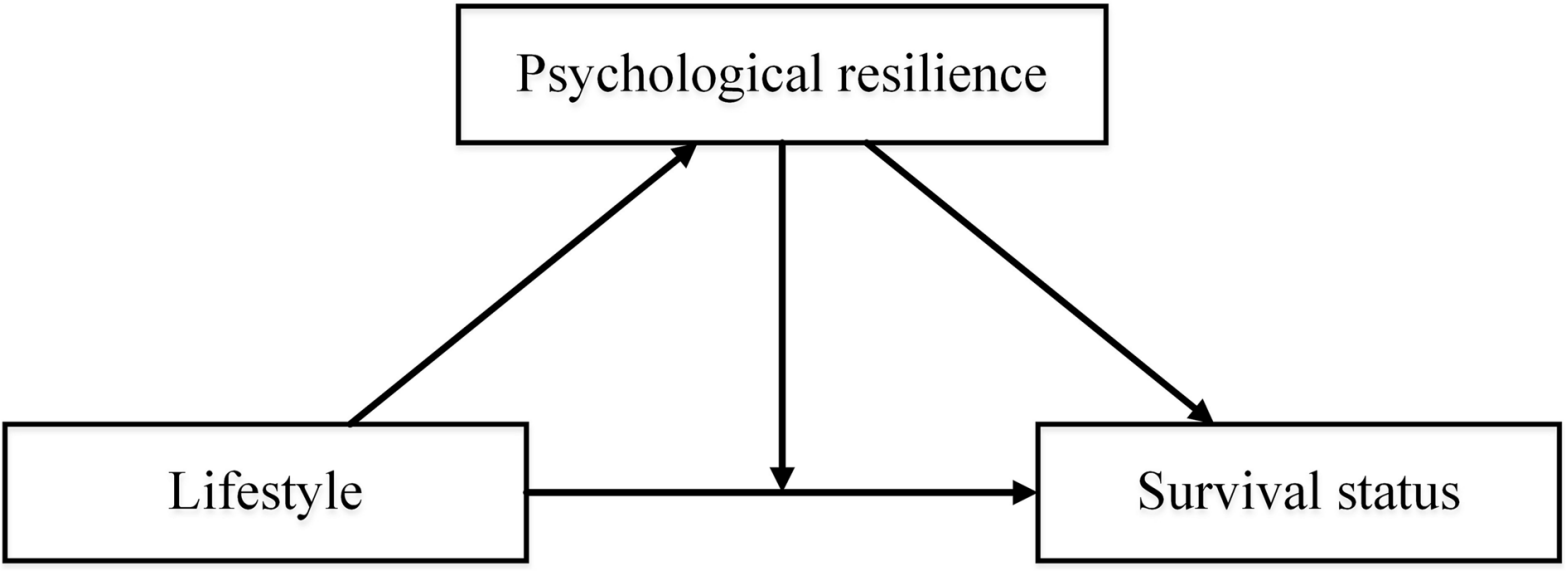
Potential study model based on hypotheses.

In recent years, as China’s urbanization process has continued, there has been a noticeable trend of young rural residents migrating to urban areas, while the number of oldest-old individuals residing in rural regions is steadily increasing. This has resulted in disparities in the living conditions between urban and rural oldest-old population. Urban residents have enjoyed significant advantages over their rural counterparts in terms of healthcare, public services, and infrastructure, such as facilities for physical activity (22, 23). Therefore, this study, stratified by urban and rural groups, would examine the mechanisms of lifestyle and psychological resilience on the survival of the oldest-old (Figure 1), which could provide policy-making support for improving life expectancy in China.

## 2 Methods

### 2.1 Study design and participants

The Chinese Longitudinal Healthy Longevity Survey (CLHLS) was designed to understand the healthcare needs of the older adult/adults and to provide important decision support for the process of healthy aging in China. The CLHLS was a nationwide, large-scale survey research project led by Peking University that was initiated in 1998, with follow-up surveys being conducted every two to three years. It covered half of the cities and counties in 23 provinces in China. This represented about 85% of the total population in the region in 2010, which was 1.156 billion people. The survey focused on people aged 65 and over. The information collected included socio-demographic characteristics, health conditions, lifestyle factors, social support, family structure, and economic circumstances (24).

This study used the CLHLS datasets from wave six (2008/2009, baseline) to wave nine (2017/2018). Initially, all participants from 2008 were included as the baseline. Subsequently, individuals under the age of 80 (*N* = 3,249) and those lost to follow-up (*N* = 4,455) were excluded, resulting in a final sample of 9,250 participants. Based on their household registration (hukou) status at baseline, the oldest-old were divided into three groups: city (*N* = 1,307), town (*N* = 1868), and rural (*N* = 6,075). In the Chinese context, the social differences between city and town areas are minimal, and this study combined them into the urban group.

### 2.2 Variables and measurements

#### 2.2.1 Outcome definition

The outcome variable in this study was the survival status of the oldest-old, specifically their all-cause mortality events (25, 26). Information on deaths was collected through ongoing tracking surveys, including follow-up surveys conducted in 2011, 2014, and 2018. Where death certificates were available, they were used to verify the participants’ information. In cases where death certificates were not available, information provided by the participant’s relatives was recorded (27). In this study, the survival time of the oldest-old was calculated in months, representing the time interval from the date of the survey to the recorded date of death for each participant.

#### 2.2.2 Independent variables

In this study, the lifestyle index was a key independent variable, assessed using five lifestyle factors (25): smoking, drinking, physical activity, dietary habits, and body mass index (BMI). Information on smoking, drinking, and physical activity was collected in the CLHLS questionnaire using ‘yes’ or ‘no’ responses. For example, answering ‘no’ to ‘Do you currently smoke?’ indicated a healthy lifestyle regarding smoking. Additionally, participants were asked four questions about the consumption of four types of food (vegetables, fruit, milk, and tea), with responses ranging from ‘almost every day’ to ‘not every day but at least once a week,’ ‘not every week but at least once a month,’ ‘not every month but occasionally,’ and ‘rarely or never.’ A healthy dietary habit was defined if a participant chose the first two options for at least two of the four food types. Furthermore, we calculated the BMI by dividing weight (in kilograms) by the square of height (in meters), expressed as kilograms per square meter. A BMI between 18.5 and 30 was considered a healthy lifestyle. Finally, for each of the five healthy lifestyle factors, a score of 1 was assigned if participants followed a healthy lifestyle, otherwise, 0 was assigned. These scores were then summed to create the lifestyle index, ranging from 0 to 5.

Psychological resilience was another important independent variable in this study. Consistent with a previous study (28), psychological resilience was assessed using a scale based on five items, such as ‘Look on the bright side of things’ and ‘Feel fearful or anxious’. Responses to each item were rated on a 5-point Likert scale, ranging from ‘always’ to ‘never.’ Reverse-coded items were transformed accordingly. The scores from the five items were summed, resulting in a total score ranging from 5 to 25, with higher scores indicating greater psychological resilience.

#### 2.2.3 Covariates

In this study, six covariates were included in the survival analysis and moderation-mediation models. These covariates were gender, age, income level, living arrangement, educational attainment, and marital status. Income level referred to the total income (in yuan) of the participants’ households in the last year, and it was categorized into three levels: <10,000, 10,000–30,000, and > 30,000 yuan. The living arrangement included three categories: living with household member(s), living alone, and living in an institution. Educational attainment was measured by the number of years of schooling of the respondents and, due to the majority having no formal education, included two categories: illiterate and non-illiterate. Marital status was divided into three groups: married, unmarried, and widowed.

### 2.3 Statistical analysis

If the variables had an approximately normal distribution, we described them using mean ± standard deviation (SD). Categorical variables were presented by frequencies and percentages (%). We calculated the mean survival time for different lifestyle choice and estimated their corresponding 95% confidence intervals (CI).

Besides, to depict the survival trends of the oldest-old clearly, we conducted a Kaplan–Meier survival analysis. The Log-rank test was employed to assess whether the survival times of the oldest-old individuals with different lifestyles were equivalent. Additionally, after adjusting for covariates, we performed a Cox regression model to explore risk factors associated with survival in the oldest-old. Results were reported in terms of Hazard Ratios (HR) along with their 95% CIs. We also tested the assumptions of proportional hazards and found that the conditions for using Cox were completely satisfied.

In this study, considering the necessity to validate the mediating and moderating effects of psychological resilience, we employed the statistical analysis package ‘med4way.’ This package could be implemented using Stata software and was suitable for various types of outcomes and mediating effects. In this study, the survival status was the dependent variable, estimated using Cox regression, while the mediating variable was psychological resilience, estimated using linear regression. The ‘med4way’ command decomposed the total effect into four components: no mediation or interaction, interaction only, both mediation and interaction, and mediation only. The focus of this study was the only mediating and only interacting effects. For further details about the ‘med4way’ method, please refer to previous study (29).

Stata 14.0 MP version (Stata Corp LP, College Station, TX, United States) was used in data cleaning and statistical analysis in this study. All statistical tests were two-sided, and the *p-value* < 0.05 was considered statistically significant.

## 3 Results

### 3.1 Characteristics of the oldest-old at baseline

The participants had an average age of 93.07 (SD = 7.39) years. There were 5,685 (61.46%) females, with the proportion in urban areas (33.23%) being approximately half that of rural areas (66.77%). A total of 6,730 (72.76%) of the oldest-old were illiterate, and the illiteracy rate was higher in rural areas compared to urban populations. Additionally, 7,698 (83.22%) of the oldest-old were either unmarried or widowed, with the rural population approximately twice the size of the urban in this category. More than half of the oldest-old had a household annual income of less than 10,000 yuan. The majority of the oldest-old (82.79%) chose to live with household members. The most common lifestyle index among the oldest-old was index = 3, accounting for 38.54%. Detailed participant characteristics were in Table 1.

**Table 1:** Characteristics of different residence of the oldest-old at baseline.

Variables	Total N (%)/Mean ± SD	Urban N (%)/Mean ± SD	Rural N (%)/Mean ± SD
Age, Mean ± SD	93.07 ± 7.39	93.06 ± 7.25	93.08 ± 7.47
Gender			
Male	3,565 (38.54)	1,286 (36.7)	2,279 (63.93)
Female	5,685 (61.46)	1889 (33.23)	3,796 (66.77)
Illiteracy			
Yes	6,730 (72.76)	2080 (30.91)	4,650 (69.09)
No	2,520 (27.24)	1,095 (43.45)	1,425 (56.55)
Marriage status			
Married	1,552 (16.78)	568 (36.60)	984 (63.40)
Unmarried/widowed	7,698 (83.22)	2,607 (33.87)	5,091 (66.13)
Income (yuan/year)			
<10,000	4,740 (51.24)	1,142 (24.09)	3,598 (75.91)
10,001 ~ 30,000	2,767 (29.91)	1,161 (41.96)	1,606 (58.04)
>30,000	1743 (18.84)	872 (50.03)	871 (49.97)
Living arrangement			
With household members	7,658 (82.79)	2,635 (34.41)	5,023 (65.59)
Alone/in an institution	1,592 (17.21)	540 (33.92)	1,052 (66.08)
Lifestyle index			
Index = 0	59 (0.64)	7 (11.86)	52 (88.14)
Index = 1	369 (3.99)	90 (24.39)	279 (75.61)
Index = 2	2,167 (23.43)	537 (24.78)	1,630 (75.22)
Index = 3	3,565 (38.54)	1,153 (32.34)	2,412 (67.66)
Index = 4	2,530 (27.35)	1,060 (41.90)	1,470 (58.10)
Index = 5	560 (6.05)	328 (58.57)	232 (41.43)

### 3.2 Survival time of the oldest-old in different lifestyles

Because there were too few participants with a lifestyle index of 0, the survival estimates for this group were not robust and would not be discussed further here. Among the oldest-old individuals who had all healthy lifestyles (Index = 5), they would have the longest survival times, averaging 59.40 (SE = 2.33) months for urban individuals and 50.08 (SE = 2.63) months for the rural. Overall, as the lifestyle index increased, their survival time tended to increase gradually. See Table 2.

**Table 2:** Survival months of the oldest-old in different lifestyle and residence.

Lifestyle index	Total		Urban		Rural	
	Mean	SE	Mean	SE	Mean	SE
Index = 0	40.93	4.50	51.86	12.24	39.13	4.69
Index = 1	37.76	1.62	37.04	3.34	37.95	1.85
Index = 2	41.66	0.79	39.58	1.53	42.31	0.92
Index = 3	41.95	0.61	39.00	1.05	42.57	0.74
Index = 4	46.74	0.76	46.47	1.15	46.94	1.02
Index = 5	55.61	1.77	59.40	2.33	50.08	2.63

Largest observed analysis time was censored, the means were underestimated.

### 3.3 Kaplan–Meier survival estimates of the oldest-old

**Figure 2:**
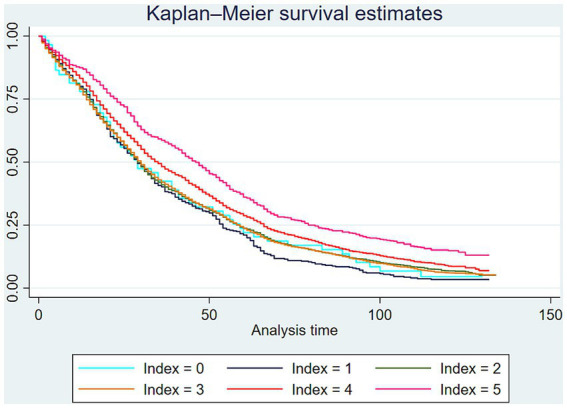
Kaplan–Meier survival estimates of the oldest-old in different lifestyle index.

Kaplan–Meier survival estimation was employed to analyze differences in the survival rates of the oldest-old across various lifestyle index groups. The results showed that the survival rate of the group of the oldest-old with index = 5 was higher than that of the other groups. However, the survival curve for the index = 0 group exhibited instability, likely due to the smaller sample size. See Figure 2.

Furthermore, the Log-rank test was conducted to examine the differences in survival rates among the oldest-old with different lifestyle index. The results indicated that the highest survival rate was observed in the index = 5 group (*p* < 0.01), followed by the index = 4 group (*p* < 0.01). See Table 3.

**Table 3:** Log-rank test for the different lifestyle index (Chi-square values).

Lifestyle index	0	1	2	3	4
1	0.52				
2	0.03	4.31^*^			
3	0.02	4.08^*^	0.13		
4	1.53	22.07^***^	20.85^***^	30.80^***^	
5	8.60^**^	53.76^***^	52.95^***^	61.85^***^	22.30***

^*^p < 0.05, ^**^p < 0.01, ^***^p < 0.001.

### 3.4 Cox regression for determinants of survival status

The Cox regression showed that for urban oldest-old, the lifestyle index served as a protective factor for survival outcomes (HR = 0.96, 95% CI: 0.93–0.98). However, this effect lost statistical significance among rural oldest-old. For all the oldest-old participants, regardless of their residence, psychological resilience played a protective role in survival outcomes (HR = 0.97, 95% CI: 0.96–0.98).

Besides, the female oldest-old exhibited a longer survival probability (HR = 0.76, 95% CI: 0.72–0.80). Living with household members was a protective factor for survival among the oldest-old (HR = 0.89, 95% CI: 0.83–0.95), but this effect did not show statistical significance in urban groups (HR = 0.94, 95% CI: 0.83–1.06). However, the survival rate significantly decreased for the unmarried or widowed (HR = 1.21, 95% CI: 1.13–1.31). Income and educational level showed no significant impact on the survival status of the oldest-old. See Table 4.

**Table 4:** Cox regression for determinants of survival of the oldest-old.

Variables	Total		Urban		Rural	
	HR	95% CI	HR	95% CI	HR	95% CI
Lifestyle index	0.96^**^	(0.93, 0.98)	0.91^***^	(0.88, 0.95)	0.98	(0.95, 1.01)
Psychological resilience	0.97^***^	(0.96, 0.98)	0.97^***^	(0.95, 0.98)	0.97^***^	(0.96, 0.98)
Female	0.76^***^	(0.72, 0.80)	0.79^***^	(0.71, 0.86)	0.74^***^	(0.69, 0.80)
Age	1.06^***^	(1.05, 1.06)	1.06^***^	(1.05, 1.07)	1.06^***^	(1.05, 1.06)
Income (10,001 ~ 30,000 yuan/year)	1.00	(0.94, 1.06)	1.01	(0.91, 1.11)	0.99	(0.92, 1.07)
Income (>30,000 yuan/year)	0.99	(0.93, 1.06)	1.02	(0.91, 1.14)	0.97	(0.88, 1.06)
Living with household members	0.89^**^	(0.83, 0.95)	0.94	(0.83, 1.06)	0.87^**^	(0.80, 0.94)
No illiteracy	0.99	(0.98, 1.00)	0.99	(0.98, 1.01)	1.00	(0.99, 1.01)
Unmarried/widowed	1.21^***^	(1.13, 1.31)	1.22^**^	(1.08, 1.38)	1.21^***^	(1.10, 1.32)

^*^p < 0.05, ^**^p < 0.01, ^***^p < 0.001.

### 3.5 Mediation and moderation role of psychological resilience on mortality

For the urban oldest-old, psychological resilience significantly mediated the effect of the lifestyle index on survival status (Coefficient = −0.006, *p* = 0.002), and it also exhibited a moderating effect between the lifestyle index and survival status (Coefficient = 0.002, *p* = 0.044). Among the rural oldest-old, psychological resilience mediated the relationship between the lifestyle index and survival status (Coefficient = −0.013, *p* < 0.001), but its moderating effect was not statistically significant (Coefficient = 0.002, *p* = 0.741). For all oldest-old, the lifestyle index had a direct impact on their survival status (Coefficient = −0.048, *p* = 0.003), and psychological resilience mediated the relationship between the lifestyle index and survival status (Coefficient = −0.013, *p* < 0.001), but its moderating effect was not significant (Coefficient = 0.005, *p* = 0.417). See Table 5.

**Table 5:** Mediation and moderation role of PR on survival of the oldest-old.

Specific effects	Coefficient	S.E.	Z	*p-value*	95% CI
*The oldest-old in urban areas*					
LI → Survival (direct effect)	−0.049	0.011	−4.49	<0.001	(−0.071, −0.028)
LI → PR → Survival	−0.006	0.002	−3.14	0.002	(−0.009, −0.002)
LI × PR → Survival	0.002	0.001	2.02	0.044	(0.001, 0.004)
*The oldest-old in rural areas*					
LI → Survival (direct effect)	−0.022	0.021	−1.08	0.282	(−0.063, 0.018)
LI → PR → Survival	−0.013	0.003	−3.94	<0.001	(−0.020, −0.007)
LI × PR → Survival	0.002	0.007	0.33	0.741	(−0.011, −0.016)
*Total of the oldest-old*					
LI → Survival (direct effect)	−0.048	0.016	−2.96	0.003	(−0.080, −0.016)
LI → PR → Survival	−0.013	0.003	−4.84	<0.001	(−0.018, −0.008)
LI × PR → Survival	0.005	0.006	0.81	0.417	(−0.007, 0.017)

LI, lifestyle index; PR, psychological resilience; S.E., standard error; CI, confidence interval. “×” meant the moderating effects, and → meant the regression path.

## 4 Discussion

Choosing healthy lifestyle is important for the oldest-old, as it can help to strengthen muscles, bones, and the cardiovascular system, slow down physical frailty, and prevent common health problems (30, 31). The oldest-old face multiple vulnerabilities such as physical function decline, social isolation, and increased caregiving needs, which can potentially lead to lots of psychological challenges (32, 33). However, there are currently few studies on the relationship between psychological resilience and survival status among the oldest-old. Stratified by urban and rural areas, this study explored the effect of lifestyle and psychological resilience on the survival status of the oldest-old. The findings could inform the development of public health projects aimed at promoting successful aging in China.

The study found that the oldest-old who followed five healthy lifestyles had the longest survival rates than that the other groups. Maintaining holistic and healthy lifestyles strengthen the function of the immune system among the oldest-old, which enhances resistant ability to infections and chronic diseases like heart disease, diabetes, and cancer (34, 35). Furthermore, the results suggested that as the lifestyle index increased, the survival rate of the oldest-old improved gradually. Verônica Colpani and colleagues explored the effects of lifestyle on mortality, and also found that adherence to more healthy lifestyles substantially declined the burden of cardiovascular disease and reduced the risk of mortality among middle-aged and older adult/adults (36). Holding more healthy lifestyle factors implies fewer health risk exposures for the oldest-old, which could result in better health status and ultimately longer life expectancy. Therefore, we suggested that the government should foster the knowledge of healthy lifestyles among the oldest-old, and increase their awareness of cultivating good living habits in daily life.

This study found that psychological resilience played a protective role in survival outcomes for the oldest-old in China. The oldest-old often face various sources of stress, such as health issues related to physical frailty, economic burdens associated with caregiving, and social isolation (37, 38). These negative events require a strong psychological resilience to adopt appropriate solutions. Higher levels of psychological resilience are associated with the generation of positive emotions and attitudes, such as optimism, hope, and satisfaction, which enable the oldest-old to quickly recover from adversities, thus enhancing their desires to survive (39). Therefore, we recommend the public enhance routine monitoring of the psychological resilience of the oldest-old, to identify any existing resilience-related issues promptly, and to implement targeted interventions to improve their psychological well-being and enhance their chances of longevity. However, this study has found that the psychological resilience of the oldest-old in rural areas did not have a direct association with their survival rate. The weak healthcare coverage, care resources, and social support network in rural areas often provide insufficient support and assistance to the oldest-old when they face difficulties (40, 41). This may weaken the impact of psychological resilience on their survival rate. Therefore, strengthening care and support is of crucial significance for the oldest-old in rural areas.

Besides, the results suggested that the female oldest-old exhibited a longer survival probability. Previous studies have shown that the life expectancy of female was higher than those of male, which was called as “male–female health-survival paradox” (42, 43). Additionally, the unmarried or widowed oldest-old showed a lower survival rate than the married ones, which was in line with previous studies (26, 44). This study also found that the rural oldest-old living with household members were more likely to be longevity, while this was not the case in urban areas. “Empty nesters” are common in rural areas and they lack long-term care from their children who are migrant workers (27). If the oldest-old in rural areas are able to live with family members, they will develop a sense of security and belonging, thus contributing to their longevity (45). Urban residents enjoy more formal community care and social services, which are greater than home care, so living with family members may not be effective in improving survival (46). Therefore, the rural communities should strengthen the provision of public service resources to meet the needs of the oldest-old, so as to promote the realization of longevity projects in China.

We further examined the mediating and moderating role of psychological resilience in the survival of the oldest-old population. For all oldest-old individuals, psychological resilience mediated the relationship between lifestyle and survival status. A healthy lifestyle can ensure that the oldest-old keep in good physical condition, avoid the multiple problems caused by illness, and create a sense of happiness and self-worth, which can elevate the determination and belief of the oldest-old to overcome difficulties and improve their survival rate (14, 47). Currently, there is a lack of attention to the lifestyles and psychological resilience of the oldest-old in China. Therefore, we recommend that the government strengthen professional education initiatives on healthy lifestyles for the oldest-old and implement timely interventions to enhance their mental well-being. For the urban oldest-old, this study found that psychological resilience significantly moderated the effect of the lifestyle on survival status. The urban oldest-old population who possess good psychological resilience, coupled with adequate social support, can have the ability to cope with difficulties, so their healthy lifestyles are more conducive to improving both quality of life and survival rates (48, 49). However, the moderating effect was not statistically significant in the rural oldest-old. This discrepancy may be attributed to the backward economic conditions, inadequate medical coverage, and limited care resources available to older adults in rural areas. Even with high psychological resilience, they still encounter substantial difficulties in terms of survival due to the constraints imposed by their living environment and available resources.

Some limitations should be mentioned in this study. Firstly, the assessment of a healthy lifestyle based on the information from five behaviors was not exhaustive. Due to constraints in the scope of CLHLS data collection, other measurements of lifestyle were not included in the study, which potentially introduced bias. Secondly, over a quarter of the participants were lost to follow-up, which may lead to bias in the results of survival estimation. Thirdly, our study primarily focused on the dependent variable of all-cause mortality and future research is warranted to estimate the specific mortality associated with lifestyle and psychological resilience. Lastly, we included only demographic and economic covariates, while other relevant covariates were omitted from the statistical model due to missing data. Therefore, future studies should consider incorporating additional control variables to enhance the robustness of the findings.

## 5 Conclusion

The oldest-old who had all five healthy lifestyles showed the longest survival rate, and as the lifestyle index increased, the survival rate of the oldest-old improved gradually. Stratified by urban and rural groups, this study showed that healthy lifestyles and psychological resilience significantly improved the survival of the urban oldest-old, while for the rural ones, the healthy lifestyles did not affect survival status. Examining the mediating and moderating effects of psychological resilience, we found that for urban oldest-old individuals, psychological resilience significantly mediated and moderated the effect of lifestyle on survival status. However, for the rural oldest-old individuals, psychological resilience could only play a mediating role in the effect of lifestyle on survival status. Consequently, we recommend that the government should intensify continuous monitoring and provide professional education on healthy lifestyles for the oldest-old, and implement timely interventions to improve their psychological resilience, which is an inevitable way to achieve longer life expectancy in China.

## Data availability statement

The original contributions presented in the study are included in the article/supplementary materials, further inquiries can be directed to the corresponding author.

## Ethics statement

The studies involving humans were approved by Duke University Health System Institutional Review Board. The studies were conducted in accordance with the local legislation and institutional requirements. The participants provided their written informed consent to participate in this study.

## Author contributions

JC: Funding acquisition, Investigation, Software, Visualization, Writing – original draft. YG: Data curation, Project administration, Supervision, Writing – original draft, Writing – review & editing. TH: Software, Visualization, Writing – review & editing. LZ: Software, Visualization, Writing – review & editing. HJ: Software, Visualization, Writing – review & editing.
